# Mesenchymal Stem Cell-Induced Doxorubicin Resistance in Triple Negative Breast Cancer

**DOI:** 10.1155/2014/532161

**Published:** 2014-07-16

**Authors:** Dar-Ren Chen, Dah-Yuu Lu, Hui-Yi Lin, Wei-Lan Yeh

**Affiliations:** ^1^Comprehensive Breast Cancer Center, Changhua Christian Hospital, Changhua 50006, Taiwan; ^2^Graduate Institute of Neural and Cognitive Sciences, China Medical University, Taichung 40402, Taiwan; ^3^School of Pharmacy, China Medical University, Taichung 40402, Taiwan; ^4^Department of Cell and Tissue Engineering, Changhua Christian Hospital, Changhua 50006, Taiwan

## Abstract

Triple negative breast cancer (TNBC) is an aggressive histological subtype with limited treatment options and a worse clinical outcome compared with other breast cancer subtypes. Doxorubicin is considered to be one of the most effective agents in the treatment of TNBC. Unfortunately, resistance to this agent is common. In some drug-resistant cells, drug efflux is mediated by adenosine triphosphate-dependent membrane transporter termed adenosine triphosphate-binding cassette (ABC) transporter, which can drive the substrates across membranes against concentration gradient. In the tumor microenvironment, upon interaction with mesenchymal stem cells (MSCs), tumor cells exhibit altered biological functions of certain gene clusters, hence increasing stemness of tumor cells, migration ability, angiogenesis, and drug resistance. In our present study, we investigated the mechanism of TNBC drug resistance induced by adipose-derived MSCs. Upon exposure of TNBC to MSC-secreted conditioned medium (CM), noticeable drug resistance against doxorubicin with markedly increased BCRP protein expression was observed. Intracellular doxorubicin accumulation of TNBC was also decreased by MSC-secreted CM. Furthermore, we found that doxorubicin resistance of TNBC was mediated by IL-8 presented in the MSC-secreted CM. These findings may enrich the list of potential targets for overcoming drug resistance induced by MSCs in TNBC patients.

## 1. Introduction

Mesenchymal stem cells (MSCs), also called multipotent mesenchymal stromal cells, are nonhematopoietic cells that reside mainly in the bone marrow and in adipose tissue [1–3]. They have stem cell-like characteristics and are able to differentiate into osteogenic, adipogenic, and chondrogenic lineages when placed in the appropriate environments [4]. MSCs are featured as plastic adherent cells that express stromal cell markers (CD73, CD105, CD44, CD29, and CD90) in the absence of hematopoietic markers (CD34, CD45, and CD14) and endothelial markers (CD34, CD31, and vWF) [5, 6]. MSCs are characteristically recruited to injured areas or hypoxic tumor microenvironments. The homing of MSCs to tumors was among the earliest phenomenon of MSC-cancer interactions to be reported [7, 8]. In the tumor microenvironment, upon interaction with MSCs, tumor cells exhibit altered biological functions of certain gene clusters. Accumulating evidence has demonstrated that MSCs play complicated roles in tumor development and progression, by increasing stemness of tumor cells, mediating tumor cell migration, promoting angiogenesis, supporting immune responses, and inducing drug resistance [9, 10]. Therefore, comprehensive knowledge on the mechanism of interaction between cancer and MSCs is critical.

Triple negative breast cancer (TNBC) is an aggressive histological subtype with limited treatment options and a worse clinical outcome compared with other breast cancer subtypes [11]. The duration of response to chemotherapeutic regimens is usually short and commonly relapses rapidly. Doxorubicin, an anthracycline antibiotic, is considered to be one of the most effective agents in the treatment of TNBC. Unfortunately, resistance to this agent is common, leading to an unsuccessful outcome in many TNBC patients. Resistance to current standard regimens limits the available options for previously treated patients to a small number of noncross resistant regimens [12]. This makes TNBC an important issue which deserves further fundamental research.

Resistance to therapy is one of the major obstacles in cancer treatment. The mechanisms involved in classic chemotherapy resistance include enhanced activity of positive regulators of cell proliferation, loss of tumor suppressors, inactivation of cell death, or enhancement of survival functions [10]. Besides the classically defined causes of drug resistance, tumor microenvironment can also promote drug resistance by preventing drugs accumulation in tumor cells [9, 13]. In some drug-resistant cells, drug efflux is mediated by adenosine triphosphate- (ATP-) dependent membrane transporters termed adenosine triphosphate-binding cassette (ABC) transporters, which can drive the substrates across biological membranes against a concentration gradient [14]. Among dozens of human ABC transporters, three well-known ABC transporters account for most of the drug resistance phenomenon, namely, ABCB1/p-glycoprotein (P-gp), ABCC1/multidrug resistance-associated protein 1 (MRP 1), and ABCG2/breast cancer resistance protein (BCRP) [14, 15]. Chemoresistance to doxorubicin may be attributed to P-gp, MRP1, or BCRP, as doxorubicin is substrate of these ABC transporters [16].

In our present study, noticeable doxorubicin resistance of TNBC was observed by exposure of TNBC to MSC-secreted conditioned medium. Therefore, the aim of this study was to investigate the underlying mechanism of doxorubicin chemoresistance induced by MSC in TNBC. Understanding the tumor-promoting factors secreted by MSCs or the mechanism activated by MSCs in tumor cells may enrich the list of potential targets for molecular therapy and overcoming tumor drug resistance in triple negative breast cancer.

## 2. Materials and Methods

### 2.1. Materials

Rabbit anti-BCRP and anti-MRP antibodies were purchased from Santa Cruz (Santa Cruz, CA). Rabbit anti-P glycoprotein was purchased from GeneTex (Irvine, CA). Anti-mouse and anti-rabbit horseradish peroxidase- (HRP-) linked antibodies were purchased from Cell Signaling (Danvers, MA). Mouse anti-*β*-actin antibody, dimethyl sulfoxide, formaldehyde, ko143, crystal violet, 3-(4,5-cimethylthiazol-2-yl)-2,5-diphenyl tetrazolium bromide (MTT), sulforhodamine B (SRB), and trichloroacetic acid were purchased from Sigma-Aldrich (St. Louis, MO). Doxorubicin was purchased from Tocris Bioscience (Minneapolis, MN).

### 2.2. Cell Culture

Human triple negative breast cancer cells MDA-MB-231 purchased from American Type Culture Collection (Manassas, VA) were maintained in L-15 medium supplemented with 10% fetal bovine serum (FBS), 100 IU/mL penicillin, and 100 mg/mL streptomycin and were incubated at 37°C without CO_2_. Human adipose-derived mesenchymal stem cells (MSC-ad) purchased from ScienCell Research Laboratories (Carlsbad, CA) were grown on poly-L-lysin coated flask (2 *μ*g/cm^2^) and maintained in mesenchymal stem cell medium (MSCM) supplemented with 5% FBS, 1% mesenchymal stem cell growth supplement (MSCGA), and 1% penicillin/streptomycin solution. MSC-ad was incubated at 37°C in a humidified incubator under 5% CO_2_ and 95% air. Confluent cultures were passaged by trypsinization.

### 2.3. Collection of Conditioned Medium from Human Adipose-Derived Mesenchymal Stem Cells (MSC-ad)

MSC-ad human adipose-derived mesenchymal stem cells were cultured on coated flasks as described above. Subconfluent culture was refreshed by fully supplemented mesenchymal stem cell medium (MSCM) and cultured for 48 hours before medium was collected as MSC-ad conditioned medium (MSC-ad CM). The conditioned medium was filtered to remove cellular materials and supernatants were aliquoted and stored at −20°C before use [17].

### 2.4. Crystal Violet Staining

Cell viability was determined by staining with crystal violet according to our previous report [18]. After the indicated period of treatment, cells were washed with PBS twice and then fixed with 12% formaldehyde. After 10 minutes incubation at room temperature, formaldehyde was aspirated and cells were air dried for 20 minutes, followed by staining with 1% crystal violet in 50% methanol for 5 minutes. Stained cells were washed with tap water and subjected to spectrophotometric quantitation (OD 540 nm) using Thermo Multiskan Spectrum plate reader.

### 2.5. MTT Assay

Live cells were measured by using the 3-(4,5-cimethylthiazol-2-yl)-2,5-diphenyl tetrazolium bromide (MTT) assay according to our previous study [19]. Culture medium was aspirated after indicated treatment and cells were washed with PBS twice. MTT solution (0.5 mg/mL in PBS) was then added in each culture well and cells were incubated at 37°C. After incubation for 1 hour, MTT solution was removed and cells were lysed by DMSO. The absorbance was measured at 550 nm by Thermo Multiskan Spectrum plate reader.

### 2.6. Sulforhodamine B (SRB) Assay

The SRB assay is based on the measurement of cellular protein content according to our previous study [20]. Culture medium was aspirated after indicated treatment and cells were fixed with 10% trichloroacetic acid for 10 minutes. 0.4% (w/v) SRB in 1% acetic acid was then added in each culture well and stained for 30 minutes. Unbound SRB was washed out by 1% acetic acid and SRB-bounded cells were dissolved by 10 mM Tris solution. The absorbance was measured at 515 nm by Thermo Multiskan Spectrum plate reader.

### 2.7. Western Blot Analysis

After washing with ice-cold PBS, cells were lysed with radioimmunoprecipitation (RIPA) assay buffer on ice for 30 minutes. After centrifugation at 14,000 g for 20 minutes, the supernatant was used for Western blot or stored at −20°C until use. Protein concentration was measured by BCA assay kit (Pierce, Rockford, IL) with BSA as standard. Equal protein was separated on SDS-polyacrylamide gels and transferred to polyvinylidene difluoride (PVDF) membranes (Millipore, Billerica, MA). The membranes were incubated for 2 hours with 7.5% dry skim milk in PBS-Tween 20 buffer to block nonspecific binding and then incubated with primary antibodies overnight at 4°C. After washing with PBS-Tween 20, the membranes were incubated with HRP-conjugated secondary antibodies for another 1 hour. The blots were visualized by enhanced chemiluminescence (ECL; Santa Cruz Biotechnology) using classic blue autoradiography film (MIDSCI, St. Louis, MO) [21]. Quantitative data were obtained using a densitometer and Image J software (National Institute of Health, Bethesda, MA).

### 2.8. Assay of Doxorubicin Efflux by Flow Cytometry

To estimate doxorubicin efflux, cells were incubated with indicated concentrations of doxorubicin for indicated time periods at 37°C avoiding light exposure. After incubation, cells were rinsed with PBS buffer twice and incubated with fresh MSCM culture medium without doxorubicin for indicated periods allowing doxorubicin efflux from cells. After the wash periods, cells were detached by trypsin and then subjected to flow cytometry. When assessing the BCRP-mediated doxorubicin efflux, Ko143 was added during doxorubicin treatment and wash periods [22, 23]. The intracellular doxorubicin content was analyzed by Cytomics FC500 flow cytometer (Ex. 488 nm, Em. 575 nm) using CXP software (Beckman Coulter).

### 2.9. Cytokine Array

Collection of conditioned medium was described above and 1 mL of sample medium was subjected to Human Cytokine Array Panel A purchased from R&D Systems (Minneapolis, MN). By following manufacturer's instruction, blots were visualized at the end by exposing membranes to autoradiography film for at least 5 minutes (MIDSCI, St. Louis, MO).

### 2.10. Statistics

Values were expressed as mean ± S.E.M. of three independent experiments (*n* = 3). Results were analyzed by student's* t*-test and significance was defined as *P* < 0.05.

## 3. Results

### 3.1. Adipose-Derived Mesenchymal Stem Cells-Secreted Conditioned Medium Reduced Doxorubicin Sensitivity in MDA-MB-231 Human Triple Negative Breast Cancer Cells

**Figure 1:**
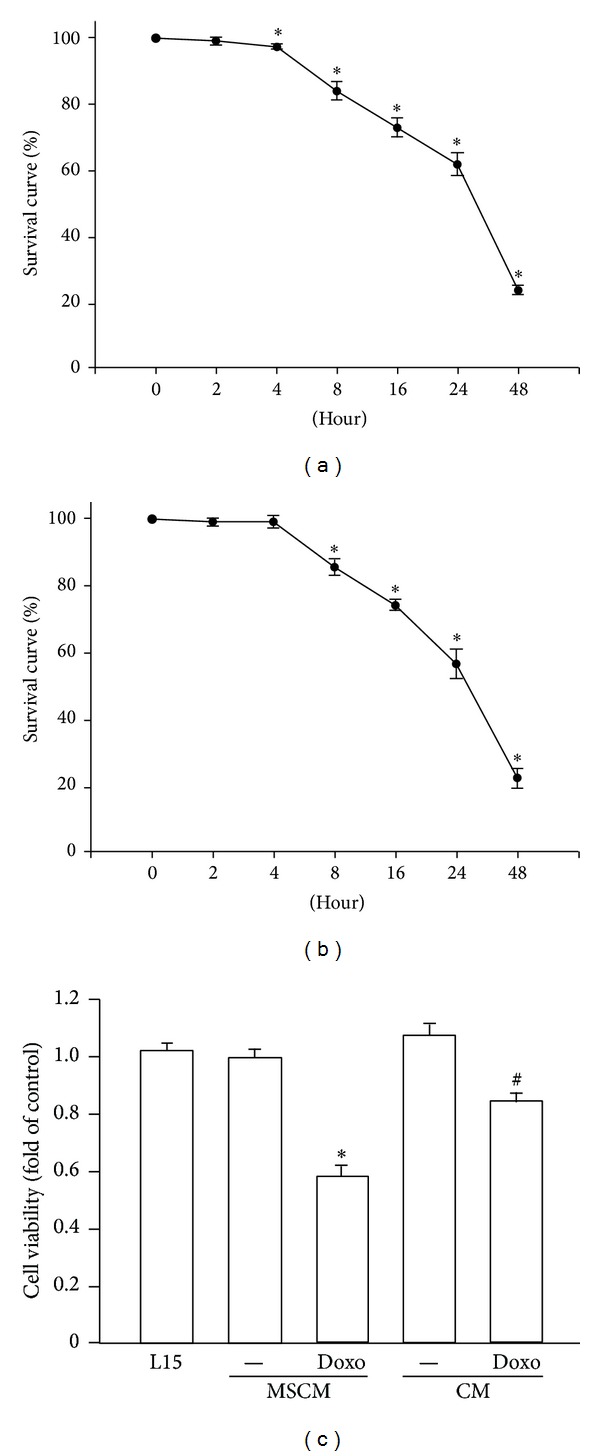
Adipose-derived mesenchymal stem cells-secreted conditioned medium induced doxorubicin resistance in MDA-MB-231 cells. Cells were treated by 200 nM doxorubicin with L15 (a) or MSCM medium (b) in a time-dependent manner and cell viability was examined by performing crystal violet staining. (c) Cells were treated by 200 nM doxorubicin with MSC-ad conditioned medium for 24 hours and cell viability was also examined by performing crystal violet staining. Graphs showed mean ± SEM of three independent experiments. *P < 0.05 to doxorubicin-untreated group; ^#^ *P* < 0.05 to doxorubicin in MSCM group. CM, conditioned medium from adipose-derived mesenchymal stem cells (MSC-ad); MSCM, mesenchymal stem cell medium (fresh MSC culture medium).

Firstly, MDA-MB-231 cells were treated by different fresh culture media (L15 or MSCM) or MSC-ad conditioned medium (CM) for 24 hours and doxorubicin was then added for another 24 hours before cell viability was assayed. As shown in Figure 1(a), 200 nM doxorubicin induced significant cell death after 4 hours' treatment on MDA-MB-231 cells in L15 medium. Similar results were obtained when MDA-MB-231 cells were in MSCM (fresh MSC culture medium) (Figure 1(b)). By the collected conditioned medium from MSC-ad, we found that MDA-MB-231 cells showed decreased cell death induced by doxorubicin. Examined by crystal violet staining, 200 nM doxorubicin in MSCM (fresh MSC culture medium) decreased cell viability to 0.58 ± 0.039-fold to control; however, doxorubicin in CM of MSC-ad only decreased cell viability to 0.84 ± 0.036-fold (Figure 1(c)). Similar results were also exhibited by MTT assay and SRB assay (data not shown). These data indicated that changing from L15 medium to MSCM medium did not affect doxorubicin sensitivity and CM from MSC-ad significantly reduced doxorubicin sensitivity in MDA-MB-231 triple negative breast cancer cells.

### 3.2. Adipose-Derived Mesenchymal Stem Cells-Secreted Conditioned Medium Increased BCRP Protein Expression and Decreased Intracellular Doxorubicin Accumulation in MDA-MB-231 Human Triple Negative Breast Cancer Cells

**Figure 2:**
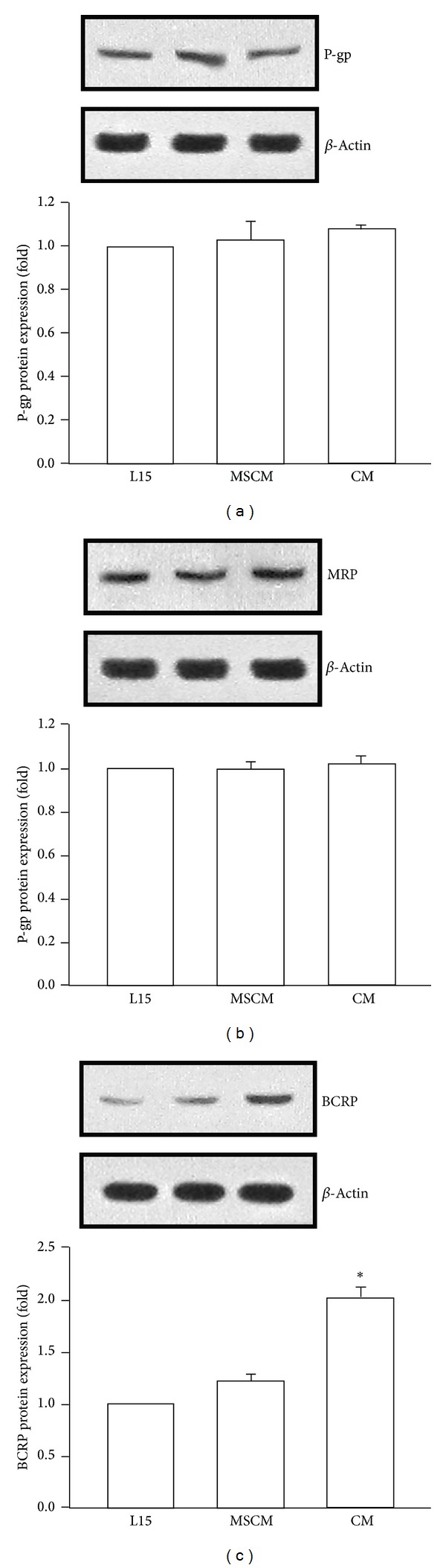
Adipose-derived mesenchymal stem cells-secreted conditioned medium increased BCRP protein expression in MDA-MB-231 cells. Cells were treated with L15 (control medium), MSCM, or MSC-ad conditioned medium for 24 hours and protein expression of (a) P-gp, (b) MRP, and (c) BCRP was examined by Western blotting. Graphs showed mean ± SEM of three independent experiments. *P < 0.05 to MSCM group. CM, conditioned medium from adipose-derived mesenchymal stem cells (MSC-ad); MSCM, mesenchymal stem cell medium (fresh MSC culture medium).

Reduced drug sensitivity may result from altered expression of ABC transporters, which can efflux substrate drugs across biological membranes against concentration gradient [14]. In order to explore the mechanism of reduced cytotoxic effect of doxorubicin caused by MSC-ad CM, we attempted to examine protein expression of ABC transporters after MSC-ad CM treatment. As shown in Figure 2, P-glycoprotein (P-gp), multidrug resistance-associated protein (MRP), and breast cancer resistance protein (BCRP) were examined. It is worth mentioning that the MRP antibody used is suitable for detection of MRP1, MRP2, and MRP3. No significant change was observed in P-gp and MRP protein expressions (Figures 2(a) and 2(b)); however, 2.01 ± 0.09-fold increase of BCRP protein expression was displayed by treating MSC-ad CM on MDA-MB-231 cells (Figure 2(c)).

**Figure 3:**
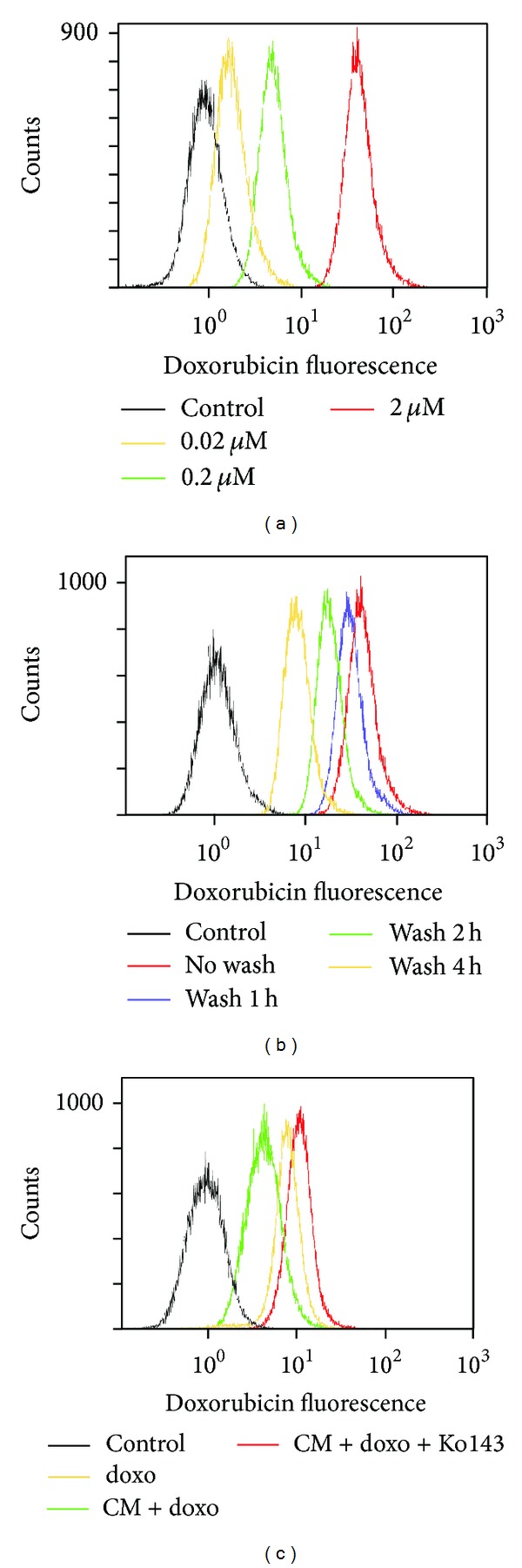
Adipose-derived mesenchymal stem cells-secreted conditioned medium decreased intracellular doxorubicin accumulation in MDA-MB-231 cells. Intracellular doxorubicin accumulation was measured by intensity of doxorubicin fluorescence using flow cytometry. (a) Cells were treated with different concentrations of doxorubicin for 1 hour without wash period. (b) Cells were treated with 2 *μ*M doxorubicin for 1 hour and then washed for 1, 2, or 4 hour(s). (c) Cells were treated with 2 *μ*M doxorubicin for 1 hour with or without conditioned medium and then washed for 4 hours. When using Ko143 as BCRP specific inhibitor, Ko143 was present during both doxorubicin-treated period and wash period. Each histogram image was a representative from three independent experiments (*n* = 3). Doxo, doxorubicin; CM, conditioned medium from adipose-derived mesenchymal stem cells (MSC-ad).

On top of the increased BCRP protein expression, we further investigated whether the intracellular doxorubicin accumulation was affected by MSC-ad CM in MDA-MB-231 cells. In order to obtain an optimal experimental condition, tests of different drug concentrations and wash periods were demonstrated. Before doxorubicin treatment, cells were refreshed in MSCM medium for 24 hours. Doxorubicin fluorescence was markedly increased dose dependently from 0.02 to 2 *μ*M (1 h) without the wash period in MDA-MB-231 cells (Figure 3(a)). Cells were incubated with doxorubicin for 1 hour avoiding light exposure and then washed and incubated with doxorubicin-free medium for indicated period to estimate doxorubicin efflux. The wash period allows doxorubicin to efflux during the period. By different duration of wash period, doxorubicin fluorescence was decreased as wash period increased from 0 to 4 hours, which suggested an increasing doxorubicin efflux and decreasing doxorubicin accumulation (Figure 3(b)). In order to explore the BCRP-mediated doxorubicin efflux, Ko143 was added as a BCRP specific inhibitor [23–25]. As shown in Figure 3(c), after pretreatment of MSC-ad CM for 24 hours, doxorubicin accumulation was decreased compared with doxorubicin in MSCM medium. Noticeably, Ko143 antagonized the effect of MSC-ad CM and resulted in an increased amount of doxorubicin accumulation significantly. These data suggested that MSC-ad CM induced BCRP protein expression without affecting P-gp and MRP and consequently decreased intracellular doxorubicin accumulation in MDA-MB-231 triple negative breast cancer cells.

### 3.3. Adipose-Derived Mesenchymal Stem Cells-Secreted IL-8 Is Responsible for Doxorubicin Resistance in MDA-MB-231 Human Triple Negative Breast Cancer Cells

**Figure 4:**
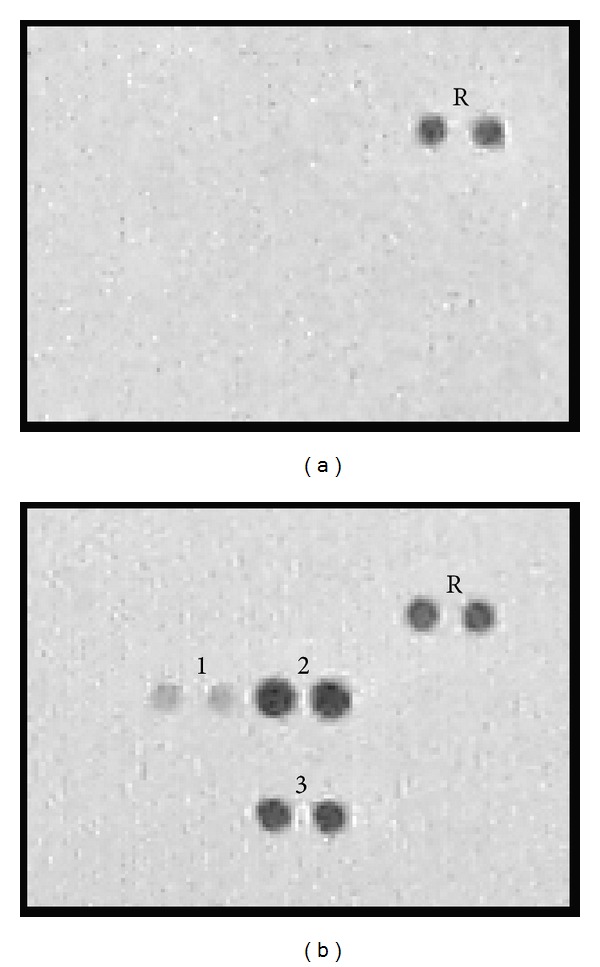
Cytokine expression of adipose-derived mesenchymal stem cells-secreted conditioned medium. Analysis of human cytokine expression of (a) MSCM (fully supplemented MSC culture medium) only and (b) MSC-ad conditioned medium by human cytokine array. R, reference spot; 1, IL-6; 2, IL-8; 3, Serpin E1.

Accumulating evidence suggests that MSCs secreted various cytokines which are associated with tumor development and progression [9, 10]. Therefore, we assumed that important cytokines had been released by MSC-ad and consequently caused the observed doxorubicin resistance in MDA-MB-231 cells. The results obtained from cytokine array analysis of MSC-ad CM and MSCM showed that IL-6, IL-8, and Serpin E1 had been secreted by MSC-ad after culturing for 48 hours. Among these cytokines, IL-8 had the most pronounced amount of secretion (Figure 4(b), dot number 2).

**Figure 5:**
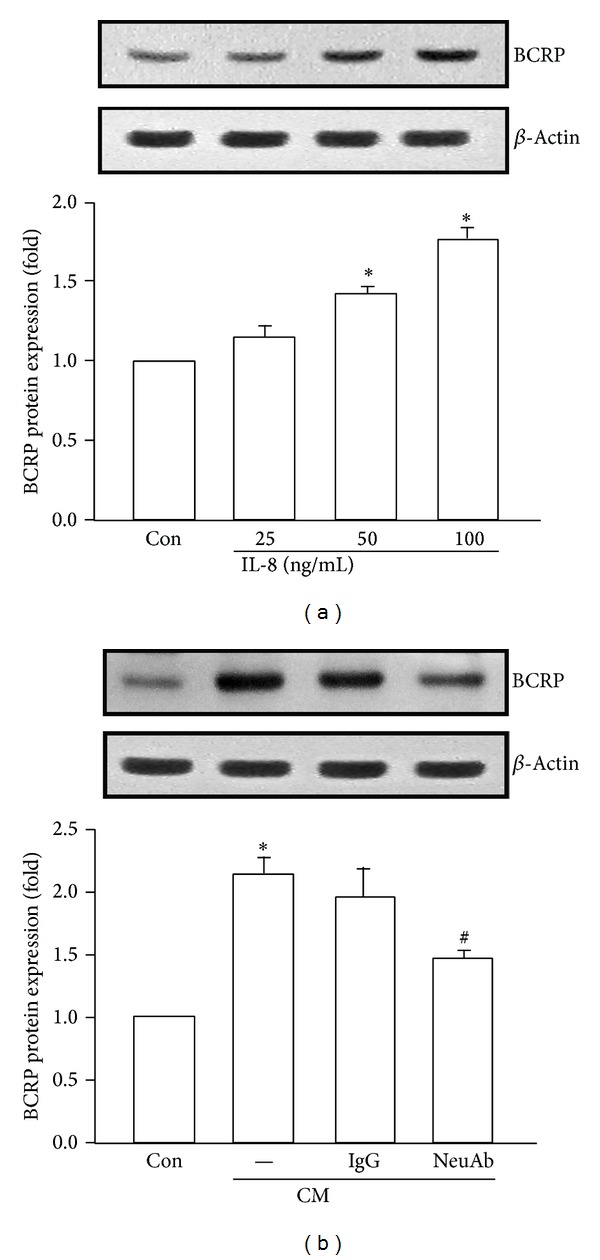
IL-8 induced BCRP protein expression in MDA-MB-231 cells. (a) Human recombinant IL-8 dose-dependently elevated BCRP protein expression after 24 hours of examination by Western blotting. (b) IL-8 neutralizing antibody (5 *μ*g/mL) antagonized MSC-ad conditioned medium-induced BCRP protein expression. IgG isotype control antibody was used as negative control. Graphs showed mean ± SEM of three independent experiments. *P < 0.05 to control group; ^#^ *P* < 0.05 to CM-treated group. CM, conditioned medium from adipose-derived mesenchymal stem cells (MSC-ad); NeuAb, IL-8 neutralizing antibody.

**Figure 6:**
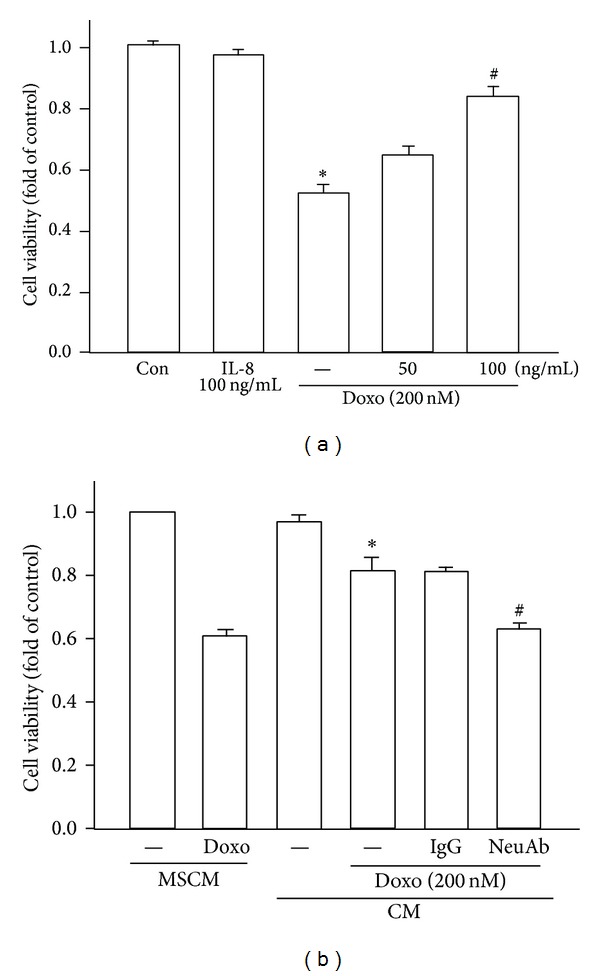
IL-8 induced drug resistance against doxorubicin in MDA-MB-231 cells. (a) Cells were treated by 200 nM doxorubicin for 24 hours with or without pretreatment of human recombinant IL-8 (50 or 100 ng/mL) for 24 hours and cell viability was examined by performing crystal violet staining. *P < 0.05 to control group; ^#^ *P* < 0.05 to doxorubicin-treated group. (b) IL-8 neutralizing antibody (5 *μ*g/mL) antagonized MSC-ad conditioned medium-induced doxorubicin (200 nM) resistance. IgG isotype control antibody was used as negative control. *P < 0.05 to MSCM with doxorubicin-treated group; ^#^ *P* < 0.05 to CM with doxorubicin-treated group. Graphs showed mean ± SEM of three independent experiments. Doxo, doxorubicin; MSCM, mesenchymal stem cell medium (fresh MSC culture medium); CM, conditioned medium from adipose-derived mesenchymal stem cells (MSC-ad); NeuAb, IL-8 neutralizing antibody.

It has been reported that IL-8 mediates drug resistance against certain anticancer agents [26, 27]. We hypothesized that IL-8 may also be responsible for the observed doxorubicin resistance in MDA-MB-231 cells. From the study of IL-8 on BCRP protein expression, we found that there was a 1.77 ± 0.07-fold increase of BCRP expression at 100 ng/mL human recombinant IL-8 stimulation and IL-8-induced BCRP expression was under a dose-dependent manner (Figure 5(a)). In order to confirm the contribution of IL-8 in MSC-ad CM in BCRP expression, IL-8 neutralizing antibody was added. MSC-ad CM-induced BCRP expression was antagonized by IL-8 neutralizing antibody (5 *μ*g/mL) from 2.13 ± 0.13-fold down to 1.47 ± 0.05 that of control (Figure 5(b)). IgG isotype control antibody was used as negative control. Furthermore, cell viability under doxorubicin treatment was also determined. IL-8 (100 ng/mL) alone did not alter cell viability; however, doxorubicin-induced cytotoxicity was reduced in the presence of 100 ng/mL IL-8 and exhibited that 0.53 ± 0.03-fold cell viability was significantly elevated to 0.85 ± 0.03 that of control (Figure 6(a)). As shown in Figure 6(b), MSC-ad CM-induced doxorubicin resistance caused cell viability to 0.82 ± 0.04 that of control. However, cell viability was markedly down to 0.63 ± 0.02 that of control in the presence of IL-8 neutralizing antibody (5 *μ*g/mL) in MSC-ad CM (Figure 6(b)). These data implicated that IL-8 secreted by MSC-ad led to increased BCRP protein expression and was responsible for reduced doxorubicin sensitivity in MDA-MB-231 triple negative breast cancer cells.

## 4. Discussion and Conclusion

In tumor microenvironment, cytokines have been secreted by cancer cells, macrophages, endothelial cells, and mesenchymal cells [28–31]. The secreted cytokine cocktail includes SDF-1, IL-1*β*, IL-3, IL-6, IL-8, TNF-*α*, NO, G-CSF, M-CSF, GM-CSF, and many others, and by various signaling pathways to protect cancer cells against chemotherapy [9]. Numerous* in vitro* and* in vivo* studies reported that cytokines are capable of modulating the expression and function of different drug transporters including P-gp, MRPs, and BCRP [26, 32, 33]. Among various types of cytokines in the tumor microenvironment, IL-8 is one of the major cytokines produced by cancer cells and stroma cells. Accumulating studies have reported that IL-8 signaling is involved in proliferation, survival, angiogenesis, and metastatic migration of cancer cells in solid tumors including ovarian, intestine, prostate, and glioma [34–36]. Increasing evidence has also suggested potential autocrine or paracrine effects of IL-8 on drug resistance in human cancers [37–39]. For example, IL-8, produced by tumor cells as an autocrine growth factor, promotes tumor growth, metastasis, angiogenesis, and chemoresistance against oxaliplatin in IL-8-overexpressing human colorectal cancer cells both* in vitro* and* in vivo* [37]. Another study has shown that autocrine production of IL-8 by ovarian cancer cells confers increased expression of apoptosis inhibitory proteins (Bcl-2, Bcl-XL, and XIAP) and P-gp, leading to cisplatin and paclitaxel resistance [38]. Furthermore, IL-8 plays a role in chemoresistance to temozolomide in melanoma side population cells [39]. However, limited studies have discussed the relation between IL-8 and breast cancer resistance protein (BCRP). BCRP, also called ABCG2, was first discovered in doxorubicin-resistant breast cancer MCF-7 cells [40]. BCRP is widely expressed in various normal tissues including mammary gland, intestine, kidney, liver, ovary, testis, placenta, endothelium, and hematopoietic stem cells [33, 41, 42]. The overexpression of BCRP is commonly found in human solid tumors such as breast, colon, ovary, and gastric cancers and accumulating evidence indicates that BCRP expression may be associated with multidrug-resistant phenotype in these cancer cells against various chemotherapeutic agents including anthracyclines, mitoxantrone, and the camptothecins by enhancing drug efflux [24, 40, 43]. In our present study, human recombinant IL-8 was found to induce BCRP protein expression, leading to doxorubicin resistance in triple negative breast cancer cells.

During tumor progression, recruitment of mesenchymal stem cells (MSCs) to tumors is reported due to the presence of soluble factors secreted in the tumor microenvironment [10]. Tumor cells secrete cytokines and growth factors to promote MSCs migration and survival [44, 45]. Hypoxic condition in the tumor microenvironment also results in the generation of cytokines and chemokines that are involved in MSCs migration to tumors [46]. When induced by soluble factors of tumor cells to migrate to the area surrounding the tumor, MSCs are involved in supporting the progression and malignant properties of tumor cells. The contribution of MSCs to drug resistance in tumor cells has also been increasingly reported. For instance, head and neck squamous carcinoma cells are resistant to paclitaxel when cocultured with bone marrow-derived MSCs [47]. MSCs can also utilize autophagy to recycle macromolecules and synthesize antiapoptotic factors to facilitate growth and survival of surrounding tumor cells [48]. In colorectal carcinoma, NRG1 released by MSCs activates PI3K/AKT pathway to stimulate growth of tumor cells [49]. Platinum-based chemotherapy in breast cancer also induces MSCs to secrete unique fatty acids that confer chemoresistance [50]. It has also been reported that MSCs are able to protect ovarian cancer cells from hyperthermia-induced cell death via SDF-1*α*/CXCR4 signaling [51]. In our presented study, conditioned medium collected from adipose-derived MSCs enhanced BCRP protein expression, leading to reduced doxorubicin sensitivity, and the secreted IL-8 is responsible for the observed phenomenon in triple negative breast cancer cells.

In some cases, patients encounter a poorer quality of life and psychological impacts after surgical removal of breast cancer, especially in young women. Hence, plastic surgery procedures for breast reconstruction concur to reduce cosmetic and psychological problems [52, 53]. In order to have a better maintenance of transplanted fat in reconstructed breast, adipose-derived mesenchymal stem cells (MSC-ad) are now added as new stem cell-enriched fat grafting techniques [54, 55]. Although breast reconstruction is safe when the remaining breast cancer cells are inactive or resting, it has not been clear whether these MSC-ad are safe for breast cancer patients because these cells may send signals that promote reactivation of the tumor cells, if there was any left [52, 53, 56–58]. According to our finding, if breast cancer recurred, MSC-ad-secreted factors may cause chemoresistance of surrounding cancer cells and place an obstacle for further treatment on breast-reconstructed patients. At the present, reconstructive therapy utilizing adipose-derived MSCs-enriched fat grafting should be considered more carefully in patients previously treated for breast cancer [59, 60].

Altogether, our study indicated that conditioned medium collected from MSC-ad increased BCRP protein expression without affecting P-gp and MRP and consequently resulted in reduced intracellular doxorubicin accumulation in MDA-MB-231 human triple negative breast cancer cells. Moreover, at least IL-8 secreted in the MSC-ad conditioned medium is responsible for the observed doxorubicin resistance. This finding provides better understanding of the role of MSCs in tumor microenvironment concerning tumor chemoresistance and shed light on discovering novel therapeutic strategies to circumvent MSCs-related drug resistance in triple negative breast cancer.

## List of Abbreviations

BCRP: Breast cancer resistance protein
CM: Conditioned medium
MRP: Multidrug resistance-associated protein
MSC-ad: Adipose-derived mesenchymal stem cell
P-gp: P-glycoprotein
TNBC: Triple negative breast cancer.
